# Early downregulation of Mcl-1 regulates apoptosis triggered by cardiac glycoside UNBS1450

**DOI:** 10.1038/cddis.2015.134

**Published:** 2015-06-11

**Authors:** C Cerella, F Muller, A Gaigneaux, F Radogna, E Viry, S Chateauvieux, M Dicato, M Diederich

**Affiliations:** 1 Laboratoire de Biologie Moléculaire et Cellulaire du Cancer, Hôpital Kirchberg, 9, rue Edward Steichen, Luxembourg 2540, Luxembourg; 2 Department of Pharmacy, College of Pharmacy, Seoul National University, 1 Gwanak-ro, Gwanak-gu, Seoul 151-742, Republic of Korea

## Abstract

Cardiac glycosides (CGs), prescribed to treat cardiovascular alterations, display potent anti-cancer activities. Despite their well-established target, the sodium/potassium (Na^+^/K^+^)-ATPase, downstream mechanisms remain poorly elucidated. UNBS1450 is a hemi-synthetic cardenolide derived from 2″-oxovorusharin extracted from the plant *Calotropis procera*, which is effective against various cancer cell types with an excellent differential toxicity. By comparing adherent and non-adherent cancer cell types, we validated Mcl-1 as a general and early target of UNBS1450. A panel of CGs including cardenolides ouabain, digitoxin and digoxin as well as bufadienolides cinobufagin and proscillaridin A allowed us to generalize our findings. Our results show that Mcl-1, but not Bcl-xL nor Bcl-2, is rapidly downregulated prior to induction of apoptosis. From a mechanistic point of view, we exclude an effect on transcription and demonstrate involvement of a pathway affecting protein stability and requiring the proteasome in the early CG-induced Mcl-1 downregulation, without the involvement of caspases or the BH3-only protein NOXA. Strategies aiming at preventing UNBS1450-induced Mcl-1 downregulation by overexpression of a mutated, non-ubiquitinable form of the protein or the use of the proteasome inhibitor MG132 inhibited the compound's ability to induce apoptosis. Altogether our results point at Mcl-1 as a ubiquitous factor, downregulated by CGs, whose modulation is essential to achieve cell death.

Cardiac glycosides (CGs) possess well-established pharmacological properties, which were more recently reconsidered for novel clinical uses. Originally prescribed to treat cardiovascular alterations, CGs also display interesting anti-cancer activities. Epidemiological studies correlated a reduced incidence of specific cancer types with the regular intake of selected CGs.^1, 2^ Preclinical *in vitro* and *in vivo* research demonstrated the ability of CGs to impact cell proliferation and survival of a variety of cancer cell models^3^ at low nanomolar concentrations. Of interest, CGs also affect cancer cell models typically resistant to canonical cytocidal agents.^4, 5, 6^ Furthermore, combinational treatments triggered synergistic effects.^7, 8, 9^

Besides apoptosis, CGs were also able to induce autophagic cell death, anoikis or immunogenic cell death.^10, 11, 12^ This heterogeneity reflects the extraordinary cytocidal potential of these compounds and reveals cross-talks existing among different cell death modalities as described for other natural compound acting as anti-cancer agents.^13^

CGs possess a steroid core structure and are sub-divided in two families depending on the chemical group on the D-ring at position 17: cardenolides (UNBS1450, ouabain, digitoxin and digoxin) contain a butyrolactone group, whereas bufadienolides (cinobufagin and proscillaridin A) exhibit an *α*-pyrone group. A sugar moiety on the A-ring of the steroid nucleus at position 3 determines pharmacokinetic and pharmacodynamic properties. Sugar moiety and lactone are in *cis*-conformation.^14^

Despite the known primary cellular target of CGs, the alpha subunit of the sodium/potassium-ATPase (Na^+^/K^+^-ATPase),^3, 14^ it was not yet possible to define a common mechanism of action. In fact, various cell death pathways are modulated by these compounds, depending on the cellular context. The dual activity played by Na^+^/K^+^-ATPases as homeostatic regulator and part of a complex signalosome^3, 4^ could explain these pleiotropic effects. The essential intracellular sensors/transducers of their anti-cancer action yet remain to be characterized.

The altered expression levels of anti-apoptotic members of the Bcl-2 (B-cell lymphoma 2) protein family is a common feature in cancer.^15^ Most cancer cell models overexpress one or more of the three major proteins: Bcl-2, Bcl-xL (B-cell lymphoma-extra-large) and Mcl-1 (myeloid cell leukemia-1). Discovered as crucial modulators of apoptosis, anti-apoptotic Bcl-2 proteins emerged more recently as important modulators of other essential cancer processes, including cell cycle, autophagy or cell metabolism.^16^ This aspect underlines the importance to target these proteins to further improve existing anti-cancer therapies. Mcl-1 became one of the most investigated members of the Bcl-2 family but the poor availability of specific Bcl-2 family inhibitors specifically targeting this protein hindered improved treatment protocols especially of Mcl-1 overexpressing and chemoresistant cancer types.^17, 18^

Despite many studies studying biological effects of CGs, only a limited number investigated their effect on Bcl-2 proteins. In a previous study, we characterized the cytostatic and pro-apoptogenic activity of the hemi-synthetic cardenolide UNBS1450 on human hematopoietic cancer cells.^19^ This compound is derived from 2″-oxovorusharin extracted from the plant *Calotropis procera* and is very effective against many cancer models while presenting excellent differential toxicity towards healthy cell types.^20, 21^

We prevously described an early downregulation of Mcl-1 without affecting Bcl-2 levels in line with results obtained with ouabain,^22^ digitoxin^23^ or selected bufadienolides.^9, 24^ Moreover Mcl-1 downregulation is a prerequisite for the induction of cell death mechanisms or the sensitization by other treatments like TRAIL (TNF-related apoptosis-inducing ligand).^9, 22, 23, 24^

Taken together, these findings suggest Mcl-1 as a common intracellular target modulated by CGs. In this study, we challenged this hypothesis by studying the modulatory effects of a panel of CGs, including both cardenolides (UNBS1450, ouabain, digitoxin and digoxin) and bufadienolides (cinobufagin and proscillaridin A) on Mcl-1 with a panel of human adherent and non-adherent cancer cell models of different origins. We show that all compounds share the common ability of modulating Mcl-1 but not Bcl-xL and Bcl-2. Taking UNBS1450 as model compound, we found that decreased Mcl-1 levels are essential for cell death induction. A microarray analysis confirmed the poor effects of UNBS1450 on transcription in general. Mcl-1 downregulation clearly occurred at the level of protein expression and required proteasome degradation but not caspases nor the BH3-only protein NOXA.

Altogether, our results indicate Mcl-1 as an ubiquitous key factor, independent of the cellular context, whose degradation is an essential early step in cell death induction modulated by CGs.

## Results

### Mcl-1 is an early and ubiquitous target of cardiac glycosides

**Figure 1:**
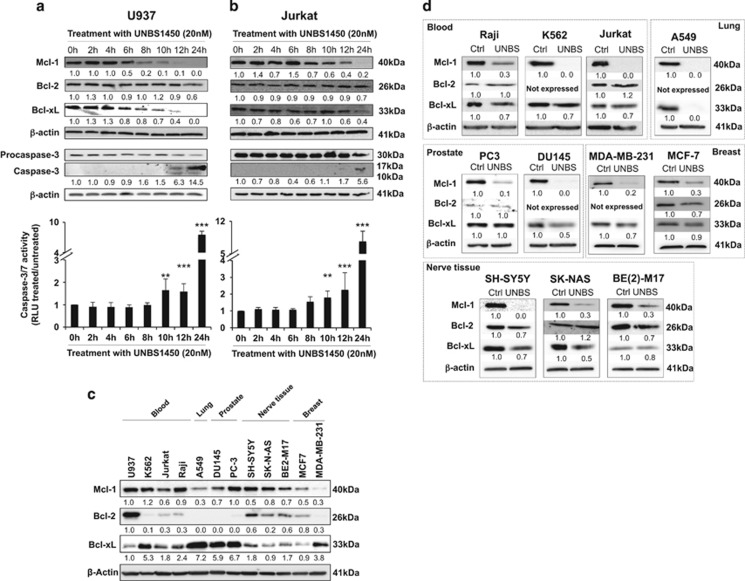
Mcl-1 protein is early and ubiquitously downregulated by UNBS1450 in human cancer cells. Kinetic analysis by western blot of Mcl-1, Bcl-xL and Bcl-2 protein expression in U937 (**a**) and Jurkat (**b**) cells following the treatment with UNBS1450 (20 nM). In parallel, caspase activation was assayed by analysis of caspase-3 cleavage and confirmed by caspase-3/7 activity assay (bottom panels). (**c**) Western blot analysis of the levels of basal expression of Mcl-1, Bcl-2 and Bcl-xL proteins in different human cell lines. (**d**) Comparative western blot analysis of Mcl-1, Bcl-2 and Bcl-xL expression after UNBS1450 treatment at the corresponding IC50 values for 24 h. One of three independent experiments is shown for the western blot analysis. Values are the mean of three independent experiments +/− S.D. Statistical analysis was performed with one-way ANOVA test (*post-hoc* test: Sidak). Significance is reported as: **P<0.01, ***P<0.001 respect control values

We analyzed the impact of the hemi-synthetic cardenolide UNBS1450 on the expression levels of the three major anti-apoptotic Bcl-2 family proteins in U937 and Jurkat cells (Figures 1a and b). Mcl-1 was the earliest downregulated protein, followed by Bcl-xL; Bcl-2 was not affected. Caspase activation took place at late treatment times in both instances. To confirm Mcl-1 as a general target of UNBS1450, we tested this compound on other human cancer cell lines (lung, prostate, breast, blood and nerve tissue) (Figure 1c). The compound was active at low nanomolar concentrations already after 24 h of treatment (Table 1). UNBS1450, used in each cell line at the corresponding IC50 concentration, systematically triggered Mcl-1 downregulation. Bcl-xL modulation depended on the cellular context. Bcl-2 levels were not at all or barely changed, whenever the cells expressed this protein (Figure 1d).

**Figure 2:**
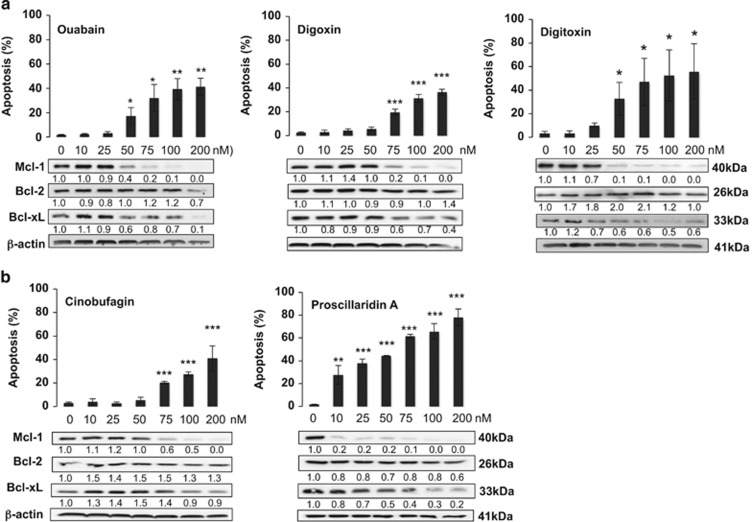
Mcl-1 is a common target of cardiac glycosides. U937 cells were treated for 24 h with the indicated concentrations of (**a**) the cardenolides ouabain, digitoxin and digoxin or (**b**) the bufadienolides cinobufagin and proscillaridin A. Apoptosis was quantified by analysis the nuclear morphology. Values are the mean of three independent experiments +/− S.D. The levels of the three major anti-apoptotic Bcl-2 proteins were analyzed in parallel by western blot. One of three independent experiments is shown. Statistical analysis was performed with one-way ANOVA test (*post-hoc* test: Sidak). Significance is reported as *P<0.01, **P<0.01, ***P<0.001 respect control values

Next, to generalize this property of CGs, we investigated the effects of three other cardenolides (ouabain, digoxin and digoxin) and two bufadienolides (cinobufagin and proscillaridin A) on Bcl-2 family protein expression and cell death induction. All CGs induced apoptosis in a dose-dependent manner between 10 and 200 nM (Figures 2a and b). We observed strongly decreased Mcl-1 expression at low apoptosis-inducing concentrations: treatment with 50 nM was generally sufficient to abrogate Mcl-1 protein expression, with proscillaridin A being even active at lower concentrations (10 nM). BCL-xL downregulation was incomplete and generally required concentrations>75–100 nM, depending on the compound. The differential modulation of Mcl-1 *versus* Bcl-xL was more evident with bufadienolides compared with cardenolides. We confirmed a reduced impact of Bcl-2 protein.

Altogether, these results document a general ability of CGs to decrease Mcl-1 expression, even before apoptosis is detectable.

### The anti-cancer effects of UNBS1450 are independent of transcriptional modulation

**Figure 3:**
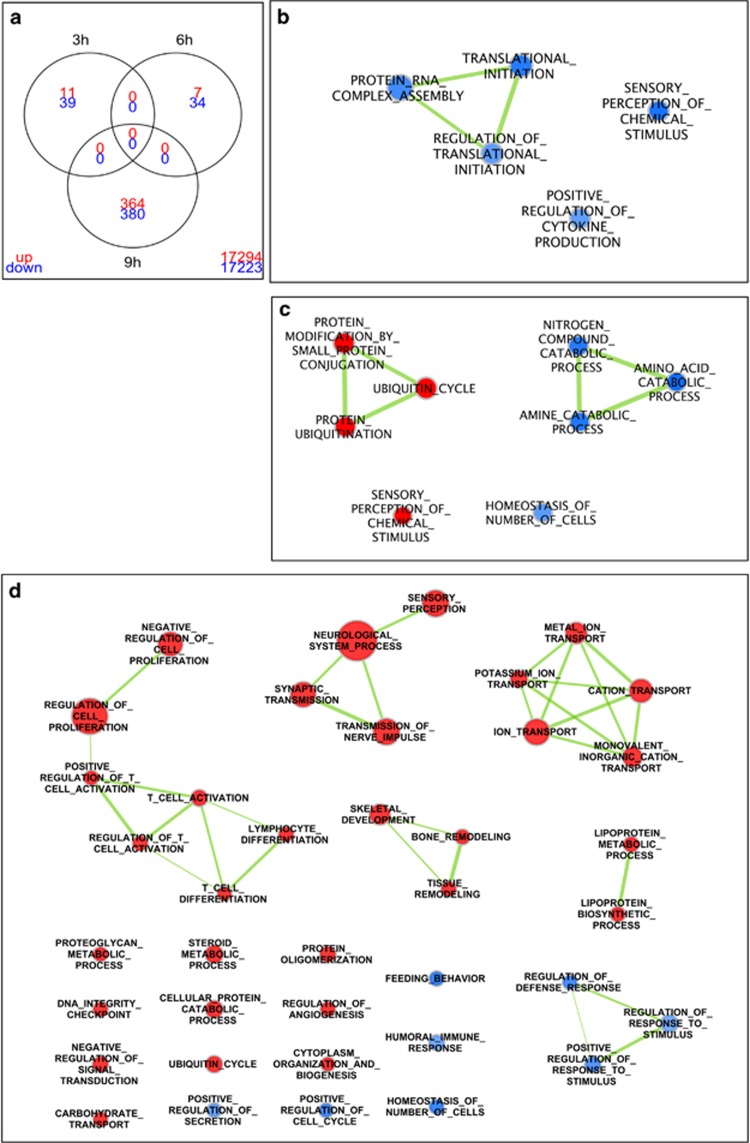
The effect of UNBS1450 on apoptosis is independent of transcriptional modulation (Microarray analysis). Venn diagram (**a**) showing the number of significant genes at each time point and overlap between gene lists. Upregulated genes (red) and downregulated genes (blue) were specified. The number at the bottom right of the figure indicated the number genes not significant in any condition (**b**–**d**). GO biological processes affected by treatment after (**b**) 3 h, (**c**) 6 h and (**d**) 9 h is reported. Upregulated processes are in red and downregulated processes are in blue. Green links indicate similarity between processes

As the time required to modulate cell death and Mcl-1 protein expression is compatible with an alteration of gene expression mechanisms, it was our first hypothesis that CGs affect transcriptional activity. We assessed the effect of UNBS1450 after 3, 6 and 9 h of treatment prior to the accumulation of apoptotic markers by a transcriptomic approach. Up- and downregulated genes are detailed in Figure 3a. Gene lists were not overlapping, meaning that no gene was differently expressed at a significant level at several time points. These results show that UNBS1450 had only a small, if any transcriptional impact at early time points. After 9 h of treatment the effect was stronger, with 744 genes significantly affected genes. Detailed results of significantly regulated genes are included in Supplementary Table S1.

We performed a functional analysis of gene lists to summarize results in biologically meaningful categories (GO biological processes). As expected from the small gene lists, only few categories were significant at 3 and 6 h (Figures 3b and c; Supplementary Table S2). After 9 h of treatment, a number of GO categories was associated with regulated genes, as shown in Figure 3d. Noteworthy, several categories were related to inflammation and cytokines. These results were in line with our previous observations showing the ability of UNBS1450 to impact important anti-inflammatory mediators such as Nuclear Factor-κB (NF-κB).^19^ Besides, transcription, RNA processing and differentiation corresponded to affected categories. Altogether we then exluded early transcriptional regulatory steps as key regulators of the molecular effects triggered by UNBS1450. We rather focussed on the effects of CGs on protein expression and turnover that we considered as more likely targets.

### UNBS1450 downregulates Mcl-1 protein levels

**Figure 4:**
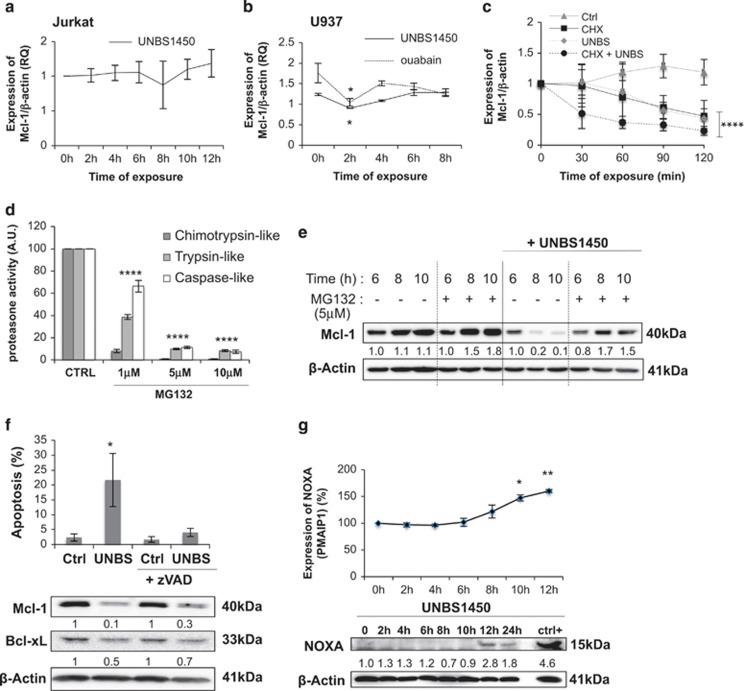
UNBS1450 downregulates Mcl-1 protein expression via a mechanism involving protein stability and the proteasome. **(a**) After treatment of Jurkat cells with UNBS1450 (20 nM) Mcl-1 mRNA expression was measured after 0, 2, 4, 6 and 8, 10 and 12 h (a time leading to Mcl-1 protein downregulation in this cell model). **(b**) Same analysis was performed on U937 cells with UNBS1450 (20 nM) or ouabain (100 nM), where Mcl-1 mRNA expression was measured after 0, 2, 4, 6 and 8 h. (**c**) Analysis of Mcl-1 protein stability. U937 cells were incubated with 20 nM UNBS1450 and after 6 h co-treated with or without 10 *μ*M cycloheximide (CHX) for further 2 h compared to untreated control cells (Ctrl). Samples were collected every 30 min and analyzed by western blot. Graph reports the quantification of Mcl-1 protein of 6 independent experiments +/−SD. (**d**) Determination of the concentration of MG132 required to inhibit the proteasome activity in U937 cells. Results are the mean of three independent experiments +/−S.D. (**e**) 5 *μ*M MG132 were selected to analyze the effect on Mcl-1 protein expression in presence/absence of UNBS1450. MG132 was added after 6 h treatment with 20 nM UNBS1450. Samples were collected after 2 h for western blot analysis of Mcl-1. (**f**) Effects of the pan-caspase inhibitor (zVAD; 50 *μ*M) on apoptosis (top) and Mcl-1/Bcl-xL expression (bottom) after 12 h treatment with UNBS1450. (**g**) Analysis of the impact of UNBS1450 on NOXA expression at mRNA and protein levels. U937 cells treated with 20-*μ*M cisplatin for 24 h were used as positive control. Western blots are representative of three independent experiments. Results are the mean of three independent experiments +/− S.D. Statistical analysis was performed by two-way ANOVA test (*post-hoc* test: Dunnett or Sidak). Significance is reported as *P<0.05, **P<0.01, ****P<0.0001. In panel (**b**), significance is reported for UNBS1450+CHX *versus* CHX or UNBS1450

Even though microarray analyses did not suggest transcriptional modulation as an early implicated mechanism, we first explored the impact of CGs on Mcl-1 mRNA expression levels by RT-PCR to further ascertain our results. Treatment of Jurkat cells with UNBS1450 did not trigger any modulation of Mcl-1 mRNA (Figure 4a). In U937 cells treated with UNBS1450 or ouabain (Figure 4b), Mcl-1 mRNA levels underwent a transient decrease prior to reaching initial levels even though Mcl-1 protein levels decreased. Altogether, these results excluded modulation of Mcl-1 mRNA levels as the cause for the observed early Mcl-1 protein decrease and prompted us to rather investigate changes at the protein level. We treated U937 cells with UNBS1450 and after 6 h we added the protein synthesis inhibitor cycloheximide (CHX). Single treatments with CHX or UNBS1450 decreased Mcl-1 protein levels similarly. The effect was amplified by a co-treatment with both compounds (Figure 4c). Proteasome inhibitor MG132 (5 *μ*M) added to U937 cells treated for 6 h with UNBS1450 abrogated the decrease of Mcl-1 protein levels (Figures 4d and e), thus indicating implication of the proteasome in the observed effect.

Bcl-2 proteins are well-known targets of caspases.^25, 26, 27^ In our instance, Mcl-1 downregulation occurred after 6 h of treatment whereas caspase activation and apoptosis happened after 10–12 h of treatment (Figures 1a and b). This different timing excludes caspases as early Mcl-1 modulators. To further confirm this hypothesis, we tested the effect of the pan-caspase inhibitor zVAD on UNBS1450-treated cells. We performed this analysis after 12 h of treatment, when caspase-3 cleavage and apoptotic cells were clearly detectable (Figure 4f). As expected, zVAD prevented caspase-3 cleavage and the accumulation of cells with apoptotic nuclear morphology. In contrast, it did not prevent Mcl-1 downregulation. Interestingly, Bcl-xL modulation also took place.

NOXA is a well-known binding partner of Mcl-1 and mediates Mcl-1 sequestration and degradation via the proteasome.^28^ We excluded any implication for this BH3-only protein in the early modulation steps of Mcl-1 by UNBS1450 as NOXA mRNA and protein only up-regulated between 10 and 12 h of treatment (Figure 4g). These results do not exclude a possible role of NOXA in late amplification loops of the apoptotic signaling.

Altogether, our results show that UNBS1450 modulated Mcl-1 expression at a post-transcriptional level, via a mechanism affecting its protein levels.

### Inhibition of Mcl-1 protein degradation protects cells against UNBS1450-induced apoptosis

**Figure 5:**
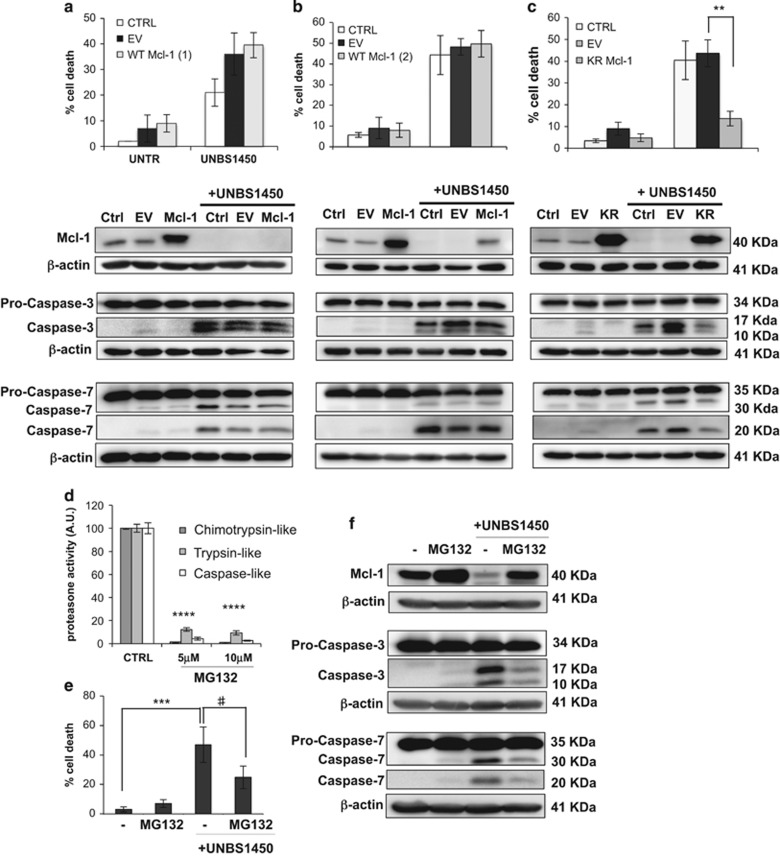
Mcl-1 downregulation regulates cytocidal effects of UNBS1450. SH-SY5Y cells were transfected and treated as reported in Material and methods with two plasmids bearing WT Mcl-1, (1) and (2) (**a** and **b**) or KR Mcl-1 plasmid (**c**). Induction of apoptosis was analyzed by nuclear morphology after 16 h UNBS1450 (25 nM) treatment. In parallel, Mcl-1 level and caspase−3/−7 cleavage were assessed by western blot. (**d**) A concentration of 5 *μ*M of MG132 was selected to study the impact on apoptosis (**e**) and (**f**) Mcl-1, caspase-3/7 cleavage in presence/absence of UNBS1450. MG132 was added after 10 h treatment with 25 nM UNBS1450, the minimum time required to induce Mcl-1 modulation in these cells (not shown), to minimize any side toxic effects of a longer exposure to the proteasome inhibitor. Western blots are representative of three independent experiments. The results are the mean of three independent experiments +/− S.D. Statistical analysis was performed by two-way ANOVA test (*post-hoc* test: Dunnett or Tukey) Significance is reported as **P<0.01, ***P<0.001, ****P<0.001 and ^#^*P*<0.05 against the respective control values

Next, we investigated whether Mcl-1 downregulation was critical for apoptosis induced by UNBS1450. To address that question, as a first strategy, we induced ectopic overexpression of the human Mcl-1 gene (Supplementary Figure 1). We used the neuroblastoma SH-SY5Y cell model, that expressed consistent levels of Mcl-1 and underwent similar modulations as U937 and Jurkat cells following treatment with UNBS1450 (Figures 1c and d). U937 and Jurkat cells models were not suitable as U937 were only poorly transfectable in our hands and Jurkat were not able to maintain WT Mcl-1 overexpression over the time of the experiment (data not shown). Transfected SH-SY5Y cells showed consistent overexpression of WT Mcl-1; after treatment with UNBS1450, however, we assisted to a severe downregulation of Mcl-1 protein, even by using two different plasmids bearing the human WT Mcl-1 gene (referred to as WT Mcl-1 (1) and WT Mcl-1 (2)).^9, 29^ Therefore, any attempt of modulating apoptosis with this approach was inconclusive (Figures 5a and b). As an alternative strategy, SH-SY5Y cells were transfected with a construct overexpressing human Mcl-1 protein with mutated ubiquitination sites (KR Mcl-1).^30^ Under these conditions, Mcl-1 overexpression was maintained even after UNBS1450 treatment and the cells overexpressing KR Mcl-1 were more resistant to UNBS1450-induced apoptosis induction compared to mock-transfected cells: we observed a reduced number of cells with fragmented apoptotic nuclei (21.8 +/− 4.4% *versus* 42.0 +/− 6.0% of apoptosis; Figure 5c) as well as the prevention of caspase-3 and -7 cleavage (Figure 5d). Transfection of U937 and Jurkat cells with KR Mcl-1 plasmid was accompanied by a lower, but consistent upregulation of Mcl-1, maintained over the time (Supplementary Figure 2). The treatment with UNBS1450, however, again almost completely abrogated Mcl-1 upregulation. This result may be a consequence of a reduced ability of these cells to be transfected. Besides, we cannot exclude the existence of additional proteasome-independent mechanisms that modulate Mcl-1 in these hematological cells.^31^ Therefore, in U937 and Jurkat, it was not possible to reliably prevent Mcl-1 downregulation to elucidate the role of this modulation in UNBS1450-induced apoptosis.

Next we addressed the question whether proteasome inhibition also protected cells against UNBS1450-induced apoptosis. Again, U937 and Jurkat cells were too susceptible to long time exposure to proteasome inhibitors and could not be used for this test. Alternatively, SH-SY5Y cells were treated for 6 h with UNBS1450 before adding 5 *μ*M of MG132. As expected, this compound prevented Mcl-1 downregulation in SH-SY5Y cells and at the same time, it strongly prevented apoptosis (Figures 5e–g).

Altogether these results show that Mcl-1 modulation is relevant for the cytotoxic potential of UNBS1450. Moreover, our results provide evidence that this modulation is essential for the anti-cancer action of CGs in general.

## Discussion

Mcl-1 modulation ubiquitously occurs in different cancer cell models, independently of origin and characteristics, following treatment with CGs. We selected the hemi-synthetic cardenolide UNBS1450 for mechanistic studies because of its favorable differential toxicity.^19^ This compound has initially been selected for its reduced affinity for the *α*-2 Na^+^/K^+^ ATPase subunit, the most important isoform expressed in cardiomyocytes,^32^ with the aim to prevent cardiac side effects.^20^ Finally, it is active at lower nanomolar concentrations than the other cardenolides, especially against drug-resistant cancer subtypes.^4, 5, 6^

In a previous study, we documented the early downregulation of Mcl-1 induced by UNBS1450 in the hematopoietic cancer cells.^19^ Here, we demonstrate that cardenolides (ouabain, digitoxin, digoxin) or bufadienolides (cinobufagin and proscillaridin A) similarly modulate Mcl-1. Interestingly, Bcl-2 is not or only modestly affected by the treatment whereas Bcl-xL modulation is not a common hallmark, but rather depending on the cellular context. Proscillaridin A in particular appears to be more differentially active against Mcl-1 *versus* Bcl-xL at lower concentrations underlining the interest of this compound for further investigations. In the future, differential molecular effects could be assessed by analysis of structure-activity relationships to suggest differential signaling patterns.

Remarkably, if there is a consistent number of papers exploring the link between the anti-cancer effects of CGs and the signaling events triggered downstream of the Na^+^/K^+^-ATPase, only a very few ones focused so far on the modulation of Bcl-2 family proteins, especially Mcl-1: the cardenolides ouabain and digitoxin were shown to downregulate Mcl-1 in lung cancer cell lines^22, 23^ whereas bufadienolides, including bufalin, bufotalin and gamabufotalin, decreased its expression in breast or adenocarcinoma cell models.^9, 24^ Altogether, Mcl-1 seems an underestimated factor while investigating the anti-cancer action of CGs.

Our studies with UNBS1450 underline the relevance of Mcl-1 downregulation to achieve apoptosis. Strategies that prevent Mcl-1 decrease, by including overexpression of a non-ubiquitinable form of Mcl-1 and inhibition of the proteasome, protected the neuroblastoma SH-SY5Y cells from UNBS1450-induced apoptosis. Vice versa, overexpression of Bcl-2 does not impact the cytocidal effect of UNBS1450 in the same cells (Supplementary Figure 3), further underlining the differential role played by these two proteins in UNBS1450-induced apoptosis.

In our hands, overexpression of WT Mcl-1 is insufficient to revert the impact of CGs on cell death. The difference in basal Mcl-1 protein levels we observed in WT Mcl-1 *versus* KR Mcl-1 SH-SY5Y overexpressing cells is likely a direct consequence of the difference in Mcl-1 protein turnover, strongly impeded in the presence of KR-Mcl-1: after 6 h of incubation with 5 *μ*M MG132 (a window of time selected to avoid cytotoxic effects of MG132 in SH-SY5Y) we observe a consistent increase in Mcl-1 levels in SH-SY5Y cells overexpressing WT Mcl-1 (4-fold with compared to basal Mcl-1 levels), therefore supporting the hypothesis that differences in the efficiency of WT *versus* KR Mcl-1 plasmids used are not the main critical reason of such a modulation (data not shown). Our results are also in line with previous reports with chemically unrelated compounds where overexpression of Mcl-1 was reduced by the treatment even in cells presenting elevated expression levels.^31, 33, 34^ Mcl-1 is controlled at many levels of its expression allowing efficient and rapid downregulation and our results demonstrate the efficiency of UNBS1450 to efficiently abrogate both constitutively and ectopically overexpressed WT Mcl-1.

One tempting hypothesis for the rapid and complete downregulation of Mcl-1 could be inhibition of its transcription or even at the level of the transcriptome. Using UNBS1450, we clearly show here that neither the transcriptome, in general, nor Mcl-1 mRNA expression are impacted by the treatment at time points compatible with the decreased protein levels. Many upstream signaling pathways converge towards the control of Mcl-1 gene transcription and its mRNA stability. Modulations involving these early steps of Mcl-1 expression were recently documented for the polyphenol quercetin.^35^

We clearly favored the idea of UNBS1450 regulating a rapid downregulation of Mcl-1 protein, without involving caspases and NOXA, both well-known modulators of Mcl-1.^18, 27^ NOXA cannot be implicated in the early phase of Mcl-1 downregulation as its upregulation after 12 h rather suggests a role in downstream amplification loops of apoptosis.

We rather suspect Mcl-1 protein degradation requiring the proteasome. Here several factors may deserve future investigations such as ubiquitin E3 ligases (as SCF^Fbw7^ and Huwe1), promoting ubiquitination, or Usp9x (ubiquitin-specific peptidase 9, X-linked) that is leading to Mcl-1 de-ubiquitination. These post-translational steps generally occur after phosphorylation of Mcl-1 and/or interaction with NOXA, most likely not a major regulator here.^36^ In addition, Mcl-1 is the one of the Bcl-2 family proteins mostly affected by metabolic alterations.^37, 38, 39^ As CGs, including UNBS1450, were shown to induce autophagy and cytostatic effects,^3, 40^ Mcl-1 modulation might become an interesting indicator of other anti-cancer (anti-metabolic?) activities of CGs, so far still to be characterized and potentially contributing to their heterogeneous biological effects. Regarding this aspect, further studies will be necessary in the future to verify the potential co-existence of ubiquitin-independent mechanisms, translational arrest or mechanisms that could contribute to Mcl-1 protein instability. The exploration of these additional areas of investigations will be very relevant in the next future. It is well known that Mcl-1 protein has a much shorter half-life than Bcl-2 and Bcl-xL proteins.^41^ This fact has recently led to consider Mcl-1 as an indicator of cell metabolism alterations through the impact of the mTOR (mammalian target of rapamycin) pathway and subsequent translational inhibition.^42^ This scenario might be also compatible with a general impact of CGs on global protein synthesis.^43, 44, 45^

Mcl-1 expression, however, does not seem to be the unique factor determining the susceptibility towards CG treatment as the comparison between basal levels of the major anti-apoptotic Bcl-2 family proteins (Figure 1c) and susceptibility to UNBS1450 in the panel of cancer cell lines examined (Table 1) is suggesting. The elucidation of the upstream modulatory mechanisms implicated, the balance with other Bcl-2 family proteins (including the pro-apoptotic Bcl-2-associated X protein (Bax) and Bcl-2 homologous antagonist killer (Bak)), as well as the potential difference generated by the cellular context remain the next challenges. The known primary target of CGs is the Na^+^/K^+^-ATPase. The effect on Mcl-1 expression is most likely the consequence of downstream signaling events.^2, 3^ Alternatively, we cannot exclude the hypothesis of a direct interaction between Mcl-1 and CGs even though, at the present level of our investigations, the timing of the observed modulation rather hints at upstream events to be involved. Our preliminary data indicate that cholesterol depletion by β-methyl-cyclodextrin efficiently prevents both Mcl-1 downregulation as well as apoptosis induced by UNBS1450 (Cerella *et al.*, manuscript in preparation). In line with our signaling hypothesis is the observation that pan-Src inhibitor also prevents UNBS1450-induced Mcl-1 downregulation. Previous findings with other CGs ^46^ generalize this observation and clearly suggest a potential ON-target effect via inhibition of Na^+^/K^+^-ATPase and Src proteins.^40^ The hypothesis of the Na^+^/K^+^-ATPase as the center of a complex signalosome remains to be further investigated but emerging evidence point at signaling cascades that are initiated downstream. The structural heterogeneity of selected CGs will then allow decoding the intricate regulatory pathways activated and explaining the selective activation of downstream kinase cascades.

In conclusion, we define here the downregulation of anti-apoptotic protein Mcl-1 as a common hallmark and essential target of CGs. Our study opens avenues for the investigation of key signaling events and the potential role of Mcl-1 as an indicator of anti-cancer activities of these compounds beyond apoptosis.

## Materials and Methods

### Cell culture

Histiocytic lymphoma U937, chronic myelogenous leukemia K562, T-cell leukemia Jurkat, Burkitt lymphoma Raji, prostate cancer PC-3 and DU145, lung adenocarcinoma A549 cell lines were purchased from DSMZ (Braunschweig, Germany). Neuroblastoma SH-SY5Y, SK-N-AS and BE(2)-M17 and breast cancer MCF-7 and MDA-MB-231 cell lines were obtained from the American Type Culture Collection (ATCC, Manassas, VA, USA). Cells were cultured in RPMI 1640 or DMEM medium (Lonza, Verviers, Belgium) supplemented with 10% (v/v) fetal calf serum (FCS; Lonza, Verviers, Belgium) and 1% (v/v) antibiotic-antimycotic (BioWhittaker, Verviers, Belgium) at 37 °C and 5% of CO2. Experiments were performed in culture medium containing 10% of FCS with cells in exponential growth phase. Cells were routinely controlled to exclude mycoplasma contamination. Non-adherent cells were seeded in fresh complete medium at concentrations of 300 000 cells/ml. After 1 h of recovery in the incubator, they were treated at the indicated concentrations. Adherent cells were seeded 36 h before treatment at concentrations of 5000 cells/ well in 96-well plates for viability and proteasome activity assays or 300 000 cells/well in 6-well plates for all the other assays.

### Compounds and treatment

All compounds were from Sigma-Aldrich (Bornem, Belgium) unless otherwise stated. UNBS1450 was a kind gift from Unibioscreen (Brussels, Belgium), prepared and stored as previously described.^19^ Ouabain, digoxin, digitoxin, cinobufagin and proscillaridin A were solubilized in dimethyl sulfoxide (DMSO) and stored at −20 °C. Cycloheximide (CHX) was diluted in sterile water at 1 mg/ml and stored at −20 °C. Cells were pre-treated for 6 h with UNBS1450 (20 nM) before adding 10 *μ*g/ml CHX. MG132 was diluted in DMSO at a stock concentration of 5 mM. For the proteasome activity assay, U937 and SH-SY5Y cells were incubated at indicated concentrations for 2 h before the measurement (see section below). For protein stability assays, U937 cells were incubated for 6 h at 20 nM UNBS1450 before adding 5 *μ*M MG132. Samples were collected for further analysis at the indicated times. For the analysis of apoptosis parameters, SH-SY5Y cells were treated with 25 nM UNBS1450 for 10 h before adding 5 *μ*M MG132 and samples were collected after 6 h. The pan-caspase inhibitor I z-VAD (OMe)-FMK (zVAD; Calbiochem, Leuven, Belgium) was diluted in DMSO at a concentration of 50 mM and added 1 h before treatment with UNBS1450 at the final concentration of 50 *μ*M.

### Cell viability assay

Cell viability was measured by using Cell Titer-Glo Luminescent Cell Viability Assay kit (Promega, Leiden, Netherlands) according to the manufacturer's instructions. The luminescence signal, proportional to the amount of ATP, was quantified by using an Orion Microplate Luminometer (Berthold, Pforzheim, Germany). Data were normalized to the control and reported as percentage of viable cells. IC50 values were estimated by treating cells with a suitable range of concentrations (from 0 to 100 nM).

### Proteasome activity assay

Proteasome activity was evaluated using Proteasome-Glo (Chymotrypsin-like/Caspase-like/Trypsin-like) cell-based assays (Promega).^47^ Cells were treated with the indicated concentrations of MG132 and the luminescence signal was quantified by using an Orion Microplate Luminometer. Cell viability was measured in parallel to exclude any bias. Data were normalized to the control and the specific proteasome activities indicated in percent.

### Analysis of apoptosis

Apoptosis was quantified by analysis of the nuclear morphology by fluorescence microscopy (Leica-DM IRB microscope, Lecuit, Luxembourg, Luxembourg), after staining cells with Hoechst 33342 (Sigma-Aldrich) The percentage of apoptotic cells was quantified as previously described by counting at least three random fields out of 100 cells.^48, 49^ For adherent cell models, trypsinization was performed before staining and analysis.

### Transfections

pCMV-HA-Mcl-1 plasmid encoding wild-type human Mcl-1, WT-Mcl-1 (1) was kindly donated by Pr. Carine Michiels (University of Namur, Belgium);^9^ pcDNA3.1-hMcl-1 was a gift from Roger Davis (Addgene plasmid 25375, Cambridge, MA, USA), indicated in the text as WT-Mcl-1 (2);^29^ pcDNA3.1 vector bearing the sequence of the human gene Mcl-1 with mutated ubiquitination sites was kindly provided by Pr Gregory J Gores (Division of Gastroenterology and Hepatology, College of Medicine, Mayo Clinic, Rochester, MN, USA).^30^ Corresponding empty control plasmids were used in parallel. Cells were transfected with Jet Prime kit (Polyplus, Ilkirch, France) according to the manufacturer's instruction. After 24 h, medium was replaced and cells were treated with the indicated compounds at the corresponding IC50 concentrations (20 nM for U937 and Jurkat cells; 25 nM for SH-SH5Y cells).

### RNA extraction and Real-Time PCR analysis

Cells were re-suspended in TRIzol (Invitrogen, Fisher Scientific, Tournai, Belgium), prior to RNA extraction, and stored at −80 °C. Two *μ*g RNA were used for reverse transcription using the SuperScriptTM III first strand synthesis system and random hexamer primers (SuperScript First-Strand Synthesis System; Invitrogen, Fisher Scientific). Real-time PCR analysis was performed using the SYBR Green PCR Master Mix (Power SYBR Green PCR Master mix 1 × , Applied Biosystems, Halle, Belgium) according to the manufacturer's protocol (7300 Real Time PCR System (Applied Biosystem). Quantification was performed in triplicate, and expression levels of Mcl-1 mRNA (sense: 5′- CCAAGGCATGCTTCGGAAA-3′, antisense: 5′-TCACAATCCTGCCCCAGTTT-3′) were normalized by using internal standards: *β*-actin (sense: 5′-CTCTTCCAGCCTTCCTTCCT-3′ antisense: 5′-AGCACTGTGTTGGCGTACAG-3′). Relative gene expression levels correspond to fold induction (RQ) compared with untreated cells.

### Extraction of cellular proteins and western blot analysis

Cells were washed with cold phosphate buffer saline (PBS); then, pellets were lysed using M-PER Mammalian Protein Extraction Reagent (Pierce, Erembodegem, Belgium) completed with a protease inhibitor cocktail (Roche, Luxembourg, Belgium), 1 *μ*M PMSF, 1 mM sodium orthovanadate, 5 mM sodium fluoride) and incubated 15 min at +4 °C in a shaking platform; then centrifuged 15 min at 15 000 at +4 °C for clarification. Twenty *μ*g of proteins were mixed with 2 × Laemmli buffer and boiled. Samples were loaded onto a sodium dodecyl polyacrylamide gel (SDS-page; stacking: 4% resolving: 10%). After migration, proteins were transferred to a polyvinylidene difluoride membrane (GE Healthcare, Roosendaal, The Netherlands), followed by blocking in PBS 0.1% Tween containing 5% milk or BSA (bovine serum albumin).

Membranes were incubated with the following primary antibodies: anti-Mcl-1 (1/1000, ON +4 °C, 5% BSA-PBS-T, Cell Signalling Biotechnology, Leiden, Netherlands, cod. 4572), anti-Bcl-xL (1/1000, ON +4 °C, 5% milk-PBS-T, BD Biosciences, Erembodegem, Belgium; cod. 610212), anti-Bcl-2 (1/2000, ON +4 °C, 5% milk-PBS-T, Calbiochem; cod. OP60), anti-Caspase-3 (1/1000, ON +4 °C, 5% milk-PBS-T, Santa Cruz Biotechnology, Boechout, Belgium; sc-56053), anti-*β*-actin (1/10000 1 h RT 5% milk-PBS-T, Sigma-Aldrich; cod. A5441). After incubation, membranes were washed with PBS-T, and incubated with the appropriate secondary antibodies anti-mouse-HRP or anti-rabbit-HRP for 1 h at RT (Santa Cruz Biotechnology or BD Biosciences). After washing, the membranes were revealed using ECL Plus Western Blotting Detection System Kit (GE Healthcare). Chemiluminescence was detected with LasMini (GE Healthcare). The bands were quantified using ImageJ, version 1.43u (from National Institutes of Health, Bethesda, MD, USA; http://rsbweb.nih.gov/ij/). Sample loading was controlled using *β*-actin.

### Microarray analysis

Treatments with 20 nM UNBS1450 were started in the exponential growth phase, 24 h after passage with 2 × 10^6^ cells. Recovery of cells was performed after 0 (no treatment), 3, 6 and 9 h, a separate control sample was performed for each time point. Total RNA was extracted from a batch of 2 × 10^6^ to 5 × 10^6^ cells by TRIzol (Invitrogen) and cleanup was performed by the RNeasy Mini Kit (Qiagen, Venlo, The Netherlands). Quantification of RNA was assessed by Nanodrop (Isogen Life science, Sint-Pieters-Leeuw, Belgium). An RNA integrity value>9 was considered acceptable (Agilent Bioanalyzer 2100, Agilent Technologies Belgium, Diegem). Microarray experiments using Agilent 4112 F Whole Human Genome Oligo microarrays (Agilent Technologies) were done according to the manufacturer's protocol with 200 ng of total RNA for the preparation of cDNA probes and Cy5- and Cy3-labeled cRNA probes (Low RNA Input Linear Amplification Kit, PLUS, Two-Color). The hybridized and washed probes on each glass slide were scanned by an Axon 4100A microarray scanner (Sunnyvale, CA, USA). Axon GenePix Pro software version 6.1 was used for feature extraction. Gene expression data were analyzed using the BioConductor^50^ package ‘LIMMA' (version 3.6.1) ^51^ in the R statistical programming environment (version 2.12.0).^52^ Spots with SNR lower than 1 or flagged as absent were down-weighted in subsequent analysis. Spots flagged as bad in every array were removed from the analysis. Pre-processing included background subtraction ‘normexp' with an offset of 50,^53^ ‘loess' within-array normalization and ‘Aquantile' between-array normalization. Entrez Gene IDs were assigned to the corresponding Agilent probe ID, using the Bioconductor annotation package ‘hgug4112a.db' version 2.4.5.^54^ Spots without Entrez Gene ID annotation were further removed. Significant spots were selected on the basis of a fold change of at least 1.3 and *P*-value cut-off of 0.05 after multiple hypothesis correction.^55^ In case of multiple spots for the same gene, only the most significant (highest F-statistic), was selected for further analysis.

### Geneset analyses

GO analysis using whole microarray results was performed using the geneset analysis algorithm ROAST,^56^ part of LIMMA package (version 3.18.13^51^) in R/Bioconductor framework (version 3.0.3),^52^ using 9999 rotations weighting genes using their average experimental expression level, as suggested in Wu *et al.*^56^ We used MsigBD ‘c5 biological process version 4.0^57^ as source, restricting the analysis to the 574 genesets of 15 genes up to 500 genes. *P*-value<0.005 was considered significant. ROAST results were visualized in Cytoscape version 2.8.2,^58^ using Enrichment Map plug-in version 1.2.^59^ We used Jaccard Overlap Combined Index and a threshold of 0.375 to link gene sets.

### Microarray data accessibility

The gene expression profiles reported in this paper have been deposited in the National Center for Biotechnology Information's Gene Expression Omnibus database. The accession number is pending.

### Statistical analysis

Data are presented as mean of at least three independent experiments±standard deviation. Significance was estimated by using one-way or two-way ANOVA tests, as further detailed in figure legends. *Post-hoc* analyses where performed using Prism 6 software, GraphPad Software (La Jolla, CA, USA). *P*-values<0.05 were considered as significant.

## Figures and Tables

**Table 1:** IC50 values were estimated after 24 h treatment with UNBS1450 on the same panel of cell models as in Figures 1c and d.

**Cell line**	**IC50 24 h (nM)**
U937	17.8±1.0
Raji	40.2±3.7
K562	32.7±3.1
Jurkat	15.6±0.7
PC3	51.0±17.0
DU145	21.0±3.0
A549	32.0±3.0
SH-SY5Y	28.4±0.4
S-K-NAS	48.7±4.9
BE(2)-M17	51.0±2.0

The impact of UNBS1450 on cell metabolism/viability was evaluated by Cell Titer-Glo assay. Values are the mean of three independent experiments±S.D.

## Abbreviations

Bax: Bcl-2-associated X protein
Bak: Bcl-2 homologous antagonist killer (Bak)
Bcl-2: B-cell lymphoma 2
Bcl-xL: B-cell lymphoma-extra large
BH: Bcl-2 Homologue
BSA: bovine serum albumin
CG: Cardiac glycoside
CHX: cycloheximide
DMSO: dimethyl sulfoxide
Mcl-1: Myeloid Cell Leukemia 1
TRAIL: TNF-Related Apoptosis Inducing Ligand
PBS: phosphate buffer saline
FCS: fetal calf serum
NF-κB: nuclear factor-κB
USP9X: ubiquitin-specific peptidase 9, X-linked

## Notes

The authors declare that they received a starting grant from the company Unibioscreen, Brussels, Belgium.
