# Supplementary figures and images for: Early downregulation of Mcl-1 regulates apoptosis triggered by cardiac glycoside UNBS1450
Source: Cell Death Dis. 2015 Jun 11;6(6):e1782–. doi: 10.1038/cddis.2015.134 (PMC4669823; doi:10.1038/cddis.2015.134)

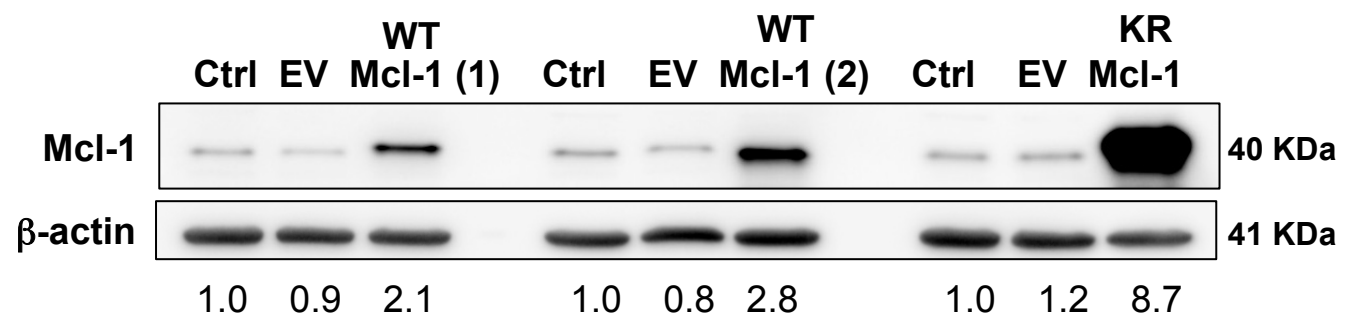

**Supplementary Figure 1**

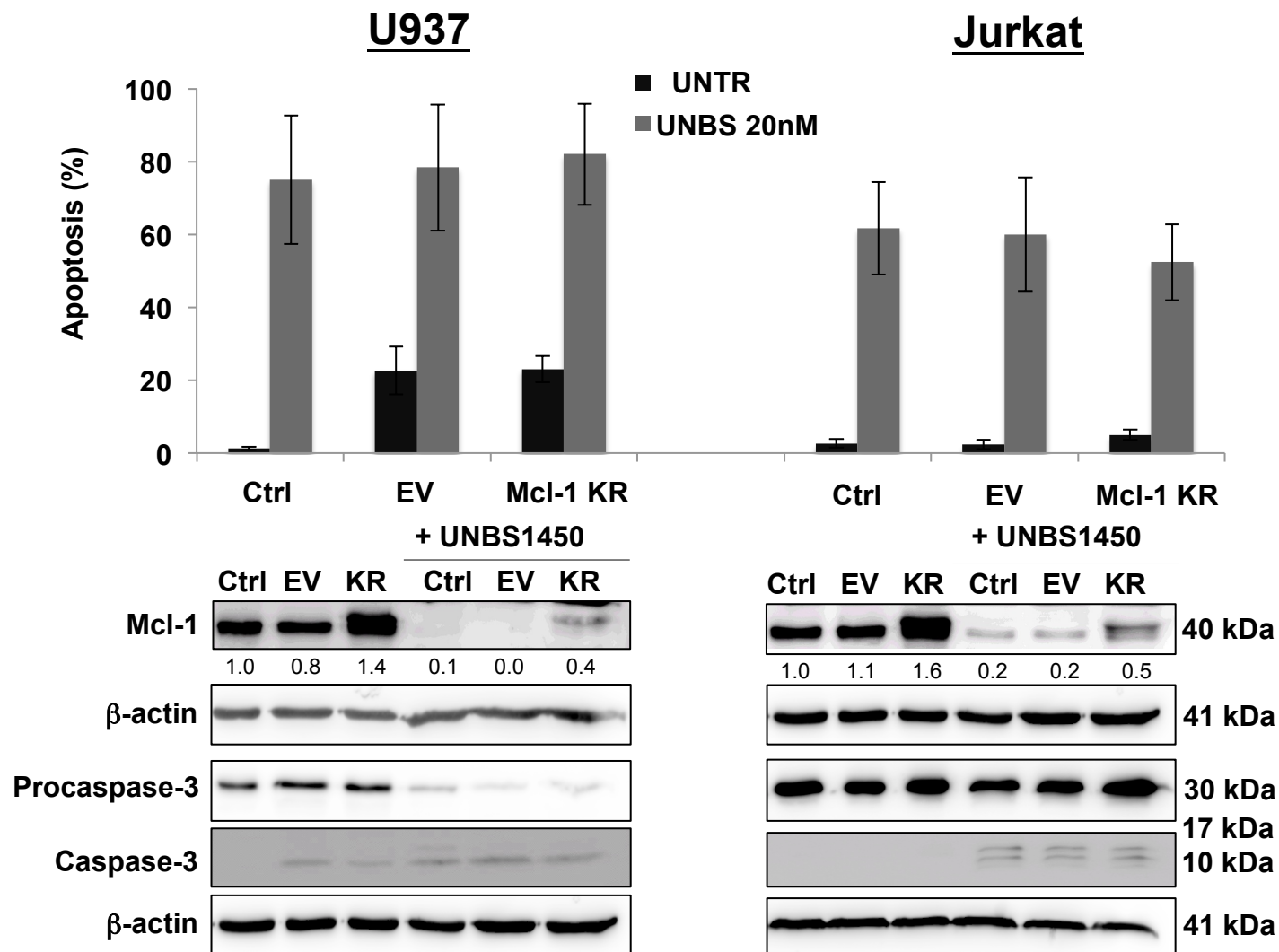

**Supplementary Figure 2**

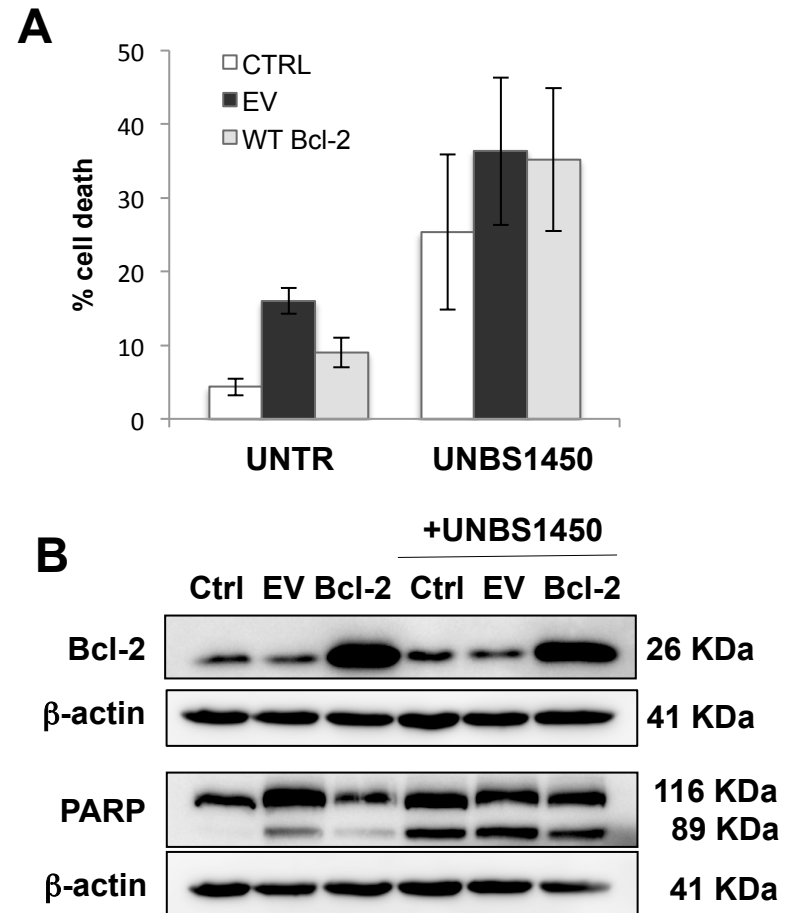

Supplementary Figure 3

Supplement: Supplementary Figure 1 [file cddis2015134x1.pdf]
