# Supplementary material for: Early downregulation of Mcl-1 regulates apoptosis triggered by cardiac glycoside UNBS1450
Source: Cell Death Dis. 2015 Jun 11;6(6):e1782–. doi: 10.1038/cddis.2015.134 (PMC4669823; doi:10.1038/cddis.2015.134)
Supplement: Supplementary Information [file cddis2015134x2.pdf]

Supplementary data

Materials and Methods

Transfections

Plasmid encoding wild-type human Bcl-2 (WT Bcl-2) was kindly donated by Pr. Patrizia Agostinis (University of Leuven, Belgium) [1]. Corresponding empty control plasmid (EV) was used in parallel. Cells were transfected with Jet Prime kit (Polyplus, Ilkirch, France) according to the manufacturer's instructions. After 24 h, medium was replaced and SH-SH5Y cells were treated with 25 nM UNBS1450. Samples were collected and used for further analysis after 16 of treatment with the compound. Antibody used for PARP-1 cleavage was from Cell Signaling Biotechnology (Leiden, Netherlands, cod. 9542).

Supplementary Figure Legends

Suppl. Figure 1. Mcl-1 expression levels in SH-SY5Y cells. SH-SY5Y cells were transfected with two different plasmids expressing WT Mcl-1 (1) and (2) (see Materials and Methods) or a KR Mcl-1-expressing plasmid. Mcl-1 levels were assessed by Western blot analysis. Ctrl represents untransfected cells. EV represents transfections with empty vector (pcDNA3). Western blots are representative of three independent experiments.

Suppl. Figure 2. Effect of UNBS1450 on KR Mcl-1 expression levels in U937 and Jurkat cells. U937 (**A**) and Jurkat cells (**B**), where transfected with KR Mcl-1 plasmid and treated with UNBS1450 (20 nM). Induction of apoptosis was analyzed by nuclear morphology (**A**) after 16 h of treatment. The results are the mean of three independent experiments +/- SD. In parallel, Mcl-1 levels and caspase-3 cleavage (**B**) were monitored by Western blot. UNTR represents untreated cells. Ctrl represents untransfected cells. EV represents transfections with empty vector (pcDNA3). One of three independent experiments with similar results is shown. Statistical analysis was performed by two-way ANOVA test (post-hoc test: Dunnett).

Suppl. Figure 3. Bcl-2 overexpression does not modulate UNBS1450-induced apoptosis. SH-SY5Y cells were transfected and treated with WT Bcl-2 plasmid.

Induction of apoptosis was analyzed by nuclear morphology (**A**) after 16 h of UNBS1450 (25 nM) treatment. The results are the mean of three independent experiments +/- SD. In parallel, Mcl-1 levels and PARP-1 cleavage (**B**) were monitored by Western blot. UNTR represents untreated cells. Ctrl represents untransfected cells. EV represents transfections with a corresponding empty vector. One of three independent experiments with similar results is shown. Statistical analysis was performed by two-way ANOVA test (post-hoc test: Dunnett).

References

1 Vantieghem A, Xu Y, Assefa Z, Piette J, Vandenheede JR, Merlevede W *et al.* Phosphorylation of Bcl-2 in G2/M phase-arrested cells following photodynamic therapy with hypericin involves a CDK1-mediated signal and delays the onset of apoptosis. *J Biol Chem*. 2002; **277**: 37718-37731.
